# Eyelash Loss: An Unusual Manifestation of Uncontrolled Hypothyroidism

**DOI:** 10.7759/cureus.59551

**Published:** 2024-05-02

**Authors:** Karen Lorena Palacios-Bayona, Catalina Tobón-Ospina

**Affiliations:** 1 Division of Endocrinology, Diabetes, Metabolism, Clínica Diagnóstica Especializada VID, Medellin, COL

**Keywords:** thyroid pathologies, overt hypothyroidism, alopecia, hair growth cycle, eyelids, eyelashes

## Abstract

Hypothyroidism commonly presents with dermatological and hair-related symptoms, although the loss of eyelashes and eyebrows is considered uncommon in clinical practice. Here, we present a case of milphosis secondary to uncontrolled hypothyroidism.

A 24-year-old female with a history of hypothyroidism following total thyroidectomy and poor medication adherence presented with significant eyelash loss, accompanied by symptoms of dysphonia, bradyphrenia, bradylalia, constipation, pronounced fatigue, and drowsiness. Physical examination revealed periorbital edema and extensive eyelash loss affecting the upper eyelids. Laboratory analysis demonstrated a markedly elevated thyroid-stimulating hormone (TSH) level of 240.8 µIU/mL (normal range 0.38 to 5.33 µIU/L), confirming severe uncontrolled hypothyroidism. Levothyroxine treatment was reintroduced, leading to complete resolution of periorbital edema and regrowth of eyelashes after 12 weeks, coinciding with improvement in TSH levels.

This clinical case adds to the limited literature on madarosis and milphosis as manifestations of hypothyroidism, emphasizing the importance of clinician awareness regarding their potential presentation in the context of the disease. Understanding these manifestations and their differential diagnoses is crucial for ensuring prompt and accurate diagnosis and treatment.

## Introduction

Primary hypothyroidism is a prevalent endocrine disorder characterized by a wide spectrum of potential manifestations, from no apparent symptoms to severe complications that can lead to multi-organ dysfunction. About 65% of patients exhibit dermatological signs such as xerosis, diffuse hair loss, facial puffiness, brittle and coarse hair, pruritus, and reduced sweating [1,2]. Among these, madarosis, or the loss of eyelashes and eyebrows, stands out as a rare manifestation, affecting approximately 1.96% of those diagnosed [1].

Madarosis divides into superciliary madarosis (loss of eyebrows) and ciliary madarosis (loss of eyelashes), commonly referred to as milphosis. The causes of madarosis are extensive and varied, rooted in autoimmune, endocrinological, infectious, genetic, neoplastic, nutritional, and traumatic sources [3-5]. It is frequently associated with several conditions including psoriasis, lupus erythematosus, eyelid trauma, blepharitis, hereditary ectodermal dysplasia syndrome, as well as side effects from certain medications like anticoagulants and antithyroid drugs, infections such as shingles and tuberculosis, cancers like sebaceous gland carcinoma, and exposure to toxic substances such as arsenic and excessive vitamin A [3-5]. Moreover, madarosis has been connected with a range of endocrine disorders including hyperthyroidism, pituitary insufficiency, hypoparathyroidism, and pituitary necrosis syndrome [6,7].

Distinguishing milphosis from other dermatological conditions impacting the eyelashes is essential for accurate diagnosis and effective management. Differential diagnoses should include trichotillomania, which is marked by compulsive hair pulling typically triggered by emotional stress, alopecia areata, characterized by patchy eyelash loss, and adnate alopecia, which involves the underdevelopment rather than the shedding of eyelashes [3].

A comprehensive medical history plays a pivotal role in diagnosis, particularly in delineating the characteristics and distribution of eyelash and other hair loss, as well as identifying potential systemic disease indicators [5]. This case report underscores the importance of considering hypothyroidism in the differential diagnosis of ciliary madarosis, emphasizing the necessity for a meticulous and vigilant diagnostic approach to effectively manage this condition.

## Case presentation

We report on a 24-year-old female with a history of surgical hypothyroidism following a total thyroidectomy for papillary thyroid carcinoma. Postoperatively, she was not treated with iodine therapy owing to a minimal recurrence risk. She was placed on a semi-annual follow-up regimen that included levothyroxine replacement therapy. However, the patient discontinued the medication without medical consultation three weeks prior to presentation, suspecting a rash was related to the therapy.

Upon presentation, she exhibited acute hypothyroidism symptoms: dysphonia, bradyphrenia, bradylalia, anxiety, constipation, significant fatigue, and drowsiness. Notably, she reported a marked increase in eyelash loss, which had started four months prior but intensified with the cessation of levothyroxine.

**Figure 1:**
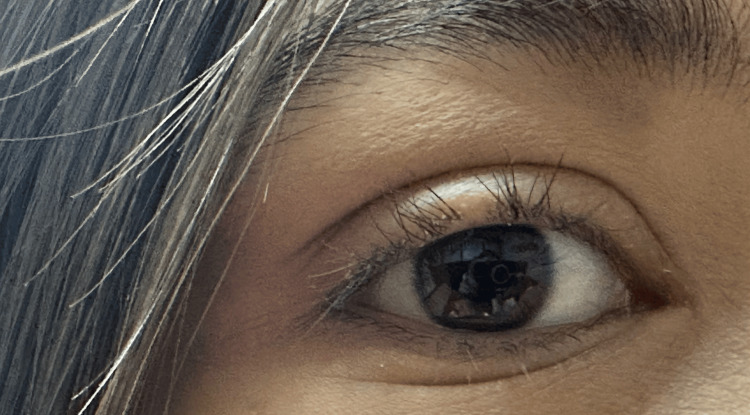
Periorbital edema and eyelash loss This image displays significant periorbital edema and eyelash loss on the upper eyelids, occurring after a three-week discontinuation of levothyroxine, with TSH levels elevated to 240.8 µIU/mL. TSH: Thyroid-stimulating hormone

The patient demonstrated stable vital signs and optimal nutritional status. Examination revealed periorbital edema and significant loss of eyelashes on the upper eyelids (Figure 1). No alopecia was noted elsewhere, and there were no rashes on the elbows or other extensor surfaces. The bilateral Achilles reflex showed a delayed relaxation phase.

Laboratory analysis disclosed a significantly elevated thyroid-stimulating hormone (TSH) level of 240.8 µIU/mL, exceeding the normal range of 0.38 to 5.33 µIU/L, confirming severe, uncontrolled hypothyroidism. Nutritional assessments ruled out deficiencies, with serum folate at 9.1 ng/mL (reference range: 4.4 to >20 ng/mL), vitamin B12 at 434 pg/mL (reference range: 197 to 771 pg/mL), ferritin at 78.9 ng/mL (reference range: 30 to 200 ng/mL), and iron at 89.7 μg/dL (reference range: 60 to 150 μg/dL).

**Figure 2:**
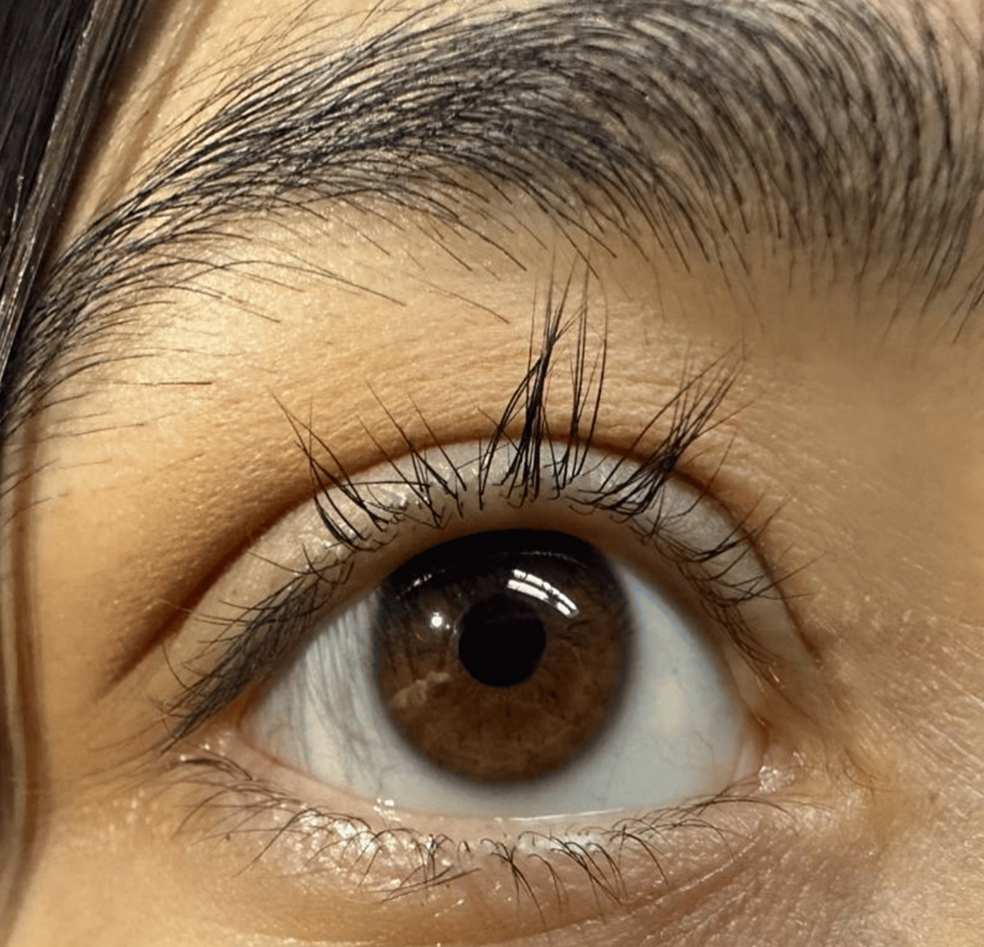
Recovery of periorbital edema and eyelash regrowth This image shows the recovery of periorbital edema and the regrowth of eyelashes after 12 weeks of levothyroxine therapy at a dosage of 1.6 mcg/kg/day, achieving a TSH level of 15 µIU/mL. TSH: Thyroid-stimulating hormone

Resumption of levothyroxine at 1.6 mcg/kg/day led to considerable clinical and biochemical improvements. A follow-up 12 weeks later showed a decreased TSH level of 15 µIU/mL. This was accompanied by the resolution of periorbital edema and partial eyelash regrowth (Figure 2). Despite persistent hair loss, no complete alopecia was observed, and osteotendinous reflexes normalized. The levothyroxine dose was further adjusted, with ongoing appointments set to fine-tune her TSH levels.

## Discussion

Ciliary madarosis, also known as milphosis, involves the loss of eyelashes and can be associated with various systemic disorders, including thyroid dysfunctions. Previous literature has documented three cases establishing a connection between ciliary madarosis and thyroid abnormalities [3,6,7], as summarized in Table 1. These cases predominantly affected women aged between 19 and 77 years, with hypothyroidism being the most commonly associated condition.

**Table 1:** Reported cases of ciliary madarosis associated with thyroid disorders TSH: Thyroid-stimulating hormone

	Case 1 [3]	Case 2 [6]	Case 3 [7]
Gender	Female	Female	Female
Age, years	25	19	77
Eyelash presentation	Multiple areas of eyelash loss on all four eyelids.	Right upper eyelid.	Multiple areas of eyelash loss on all four eyelids.
Time of eyelash loss progression	Nine years	One and a half months	Unreported.
Coexisting dermatological manifestations	Trichotillomania in eyebrows but not in eyelashes.	Without alopecia or hair loss in other areas.	Loss of hair in the eyebrows (superciliary madarosis).
Thyroid disorder diagnosed	Subclinical hypothyroidism: TSH 5.8 mIU/L (normal range 0.3 to 4.7 mIU/L) and free T4 13 pmol/L (normal range 9.1 to 23.8 pmol/L) with positive thyroid antibodies.	Hyperthyroidism.	Overt hypothyroidism: TSH 33.7 µIU/mL (normal range 0.35 to 4.94), with low free T4 and positive thyroid antibodies.
Additional coexisting medical conditions.	None	Asthma well-managed with inhalers, academic-related stress.	Hypertension and diabetes mellitus.
Time of eyelash recovery after initiating treatment for thyroid disorder.	No follow-up was obtained	Twelve weeks after the initiation of propylthiouracil	Eyelash growth was not observed despite initiating treatment with levothyroxine.

The clinical manifestations of ciliary madarosis can vary significantly, ranging from unilateral to bilateral involvement, potentially affecting all four eyelids, as observed in our patient's case. An integral aspect of the diagnostic evaluation in such instances is ruling out trichotillomania as a potential differential diagnosis, as it closely mimics the appearance of madarosis due to compulsive eyelash pulling, often triggered by psychological stress [8].

The onset of ciliary madarosis linked to thyroid disorders can vary significantly, ranging from a few months, as seen in our patient's case, to several years [3]. It's crucial to underscore that ciliary madarosis can act as a significant warning sign, prompting patients to seek medical attention [7]. Consequently, the measurement of TSH should be deemed an essential component of the evaluation in these patients, even in the absence of other apparent clinical indicators of thyroid dysfunction.

The prognosis and treatment response can vary, as noted in previous studies. In our patient's case, nearly complete restoration of eyelashes occurred within just 12 weeks of restarting levothyroxine therapy. However, it's essential to acknowledge that there are instances where treatment may not fully restore hair growth [4,7].

In summary, although uncommon, ciliary madarosis may be associated with thyroid disorders, emphasizing the importance of including TSH measurement in the differential diagnosis of patients with eyelash loss. Moreover, it could indicate uncontrolled thyroid function in those with previously diagnosed hypothyroidism, as observed in our clinical case.

## Conclusions

In summary, we present a case of severe hypothyroidism where ciliary madarosis, or eyelash loss, markedly improved within a few weeks of starting levothyroxine treatment, although thyroid function did not completely normalize. This underscores the significance of recognizing ciliary madarosis as a potential indicator of thyroid disorders, indicating its possible role as an early sign preceding other clinical symptoms. Therefore, integrating TSH measurement into the assessment of patients experiencing eyelash loss is crucial for timely detection and effective management.

## References

[1] Keen MA, Hassan I, Bhat MH (2013). A clinical study of the cutaneous manifestations of hypothyroidism in kashmir valley. Indian J Dermatol.

[2] Cohen B, Cadesky A, Jaggi S (2023). Dermatologic manifestations of thyroid disease: a literature review. Front Endocrinol (Lausanne).

[3] Jordan DR (2007). Eyelash loss. Semin Plast Surg.

[4] Mumford BP, Eisman S, Yip L (2023). Acquired causes of eyebrow and eyelash loss: a review and approach to diagnosis and treatment. Australas J Dermatol.

[5] Nguyen B, Hu JK, Tosti A (2023). Eyebrow and Eyelash Alopecia: A Clinical Review. Am J Clin Dermatol.

[6] Jordan DR, Ahuja N, Khouri L (2002). Eyelash loss associated with hyperthyroidism. Ophthalmic Plast Reconstr Surg.

[7] Matsuura H, Suganami Y (2020). Hypothyroidism, eyelash loss. Cleve Clin J Med.

[8] Venneuguès RV, Macbeth A, Levell NJ (2015). Dramatic and persistent loss of eyelashes. JRSM Open.

